# Associations between Childhood Parental Mental Health Difficulties and Depressive Symptoms in Late Adulthood: The Influence of Life-Course Socioeconomic, Health and Lifestyle Factors

**DOI:** 10.1371/journal.pone.0167703

**Published:** 2016-12-09

**Authors:** Viola Angelini, Bart Klijs, Nynke Smidt, Jochen O. Mierau

**Affiliations:** 1 Department of Economics, Econometrics and Finance, University of Groningen, Groningen, the Netherlands; 2 Statistics Netherlands, The Hague, the Netherlands; 3 Department of Epidemiology, Department of Geriatrics, University Medical Centre Groningen, Groningen, the Netherlands; 4 Department of Geriatrics, University Medical Centre Groningen, Groningen, the Netherlands; Maastricht University, NETHERLANDS

## Abstract

**Background:**

Depression among older adults (i.e., the 50+) is a major health concern. The objective of this study is to investigate whether growing up with a parent suffering from mental health problems is associated with depressive symptoms in late-adulthood and how this association is influenced by life-course socio-economic, health and lifestyle factors in childhood and late adulthood.

**Methods:**

We used life-history data from the SHARE survey, consisting of 21,127 participants living in 13 European countries. Symptoms of depression were assessed using the EURO-D scale. Parental mental health was assessed by asking respondents to report whether any of their parents had mental health problems during the respondents’ childhood. Logistic regression models were used to assess the association between parental mental health status and depression. Variables on childhood and late-life socio-economic, health and lifestyle factors were sequentially added to the model to assess the extent to which this association is influenced by life-course circumstances.

**Results:**

Individuals who were exposed during childhood to a parent with mental health problems suffered from depressive symptoms more often in late adulthood than those who were not (OR 1.76, 95% CI: 1.43–2.17). Adjustment for life-course socio-economic, health and lifestyle factors in childhood and late adulthood diminished this association to an OR of 1.54 (95% CI: 1.24–1.90) and OR of 1.45 (95% CI: 1.16–1.82), respectively.

**Conclusion:**

Our results indicate a substantial association between parental mental health problems in childhood and depression in late adulthood and that this association is partly explained by childhood as well as late adulthood socio-economic, health and lifestyle factors.

## Introduction

Depression among older adults (i.e., the 50+) is a major health concern [1]. It is estimated that 1–4% of all older adults report major depressions and 8–16% suffer from clinically significant depressive symptoms [2,3]. Worldwide, depression is the leading cause of disability for both males and females of all ages [4] and the projections are that by 2020 it will be the second contributor to the global burden of disease as measured by Disability-Adjusted Life Years (DALYs), after heart disease [5].

Psychological, psychiatric and life-course epidemiological research has shown that children of parents with mental health problems are more likely to exhibit depressive symptoms [6–8], due to the genetic component of mental health [9,10], due to the negative socio-economic and psychological consequences of living with a parent with a mental illness [11,12] and due to the interaction between the genetic and environmental components through, among others, the neurobiological functioning of genes [13]. Indeed, children sharing their childhood environment with, for instance, a depressed parent may develop depressive symptoms due to the impact of being around a depressed parent [14,15]. For instance, parents suffering from mental health problems may be less able to care for their children, causing them to grow up in adverse economic circumstances–a well-known risk factor for late-adulthood depression [14,16,17]. Using data from the 1970 British Cohort Stud, Johnson et al. (2013) find a weak positive correlation between mental of respondents and the their mothers’ 20 years earlier. This correlation was attenuated after adjusting for parental socio-economic status, child health and educational test scores.

It is not known how parental mental health problems during the childhood are associated to depression at older ages and whether this relationship is influenced by the childhood environment or by adulthood characteristics such as marital status, socio-economic conditions, health and lifestyle. These characteristics have been shown to be important risk factors for depression among older adults [9,16,18,19], and may themselves be influenced by growing up with a parent suffering from mental difficulties.

To the best of our knowledge, this is the first study to investigate whether growing up with a parent suffering from mental health difficulties is associated with depressive symptoms in late-adulthood and whether this association is influenced by life-course socio-economic, health and lifestyle factors in childhood and late adulthood. Our study focuses on a large scale (n = 21,127) cross-national sample of individuals aged 50+ from thirteen European countries–the Survey of Health Ageing and Retirement in Europe and its sub-component SHARELIFE [20].

Earlier contributions have shown that the association between current depressive symptoms and childhood parental mental health difficulties is a complex process including various potential pathways [11,21]. Firstly, a direct effect of mental health problems running through genetic factors [10]. Secondly, an indirect effect that arises from the fact that a parent with mental health difficulties potentially provides an adverse environment in which the child grows up–a known risk factor for depression later in life [14]. As outlined above, the genetic and environmental transmission channels also interact with each other [13]. Thirdly, having accumulated the set-backs provided by an adverse childhood environment in combination with the exposure to a parent with mental health difficulties, an individual may have reduced educational attainment and/or suffer from relationship instability, which again may lead to the portrayal of depressive symptoms [16,18,19].

With regard to our current study we take the above pathways as a background and hypothesize firstly, that there is a positive association between childhood parental mental health difficulties and depression in late adulthood. Secondly, we expect that the magnitude of the association declines if we take into account that parental mental health problems are themselves associated to adverse childhood circumstances. If so, this indicates that some of the association is not due to parental mental health problems as such but due to factors correlated with mental problems. Thirdly, we anticipate that the association further declines when we take into account the attenuating role of, amongst others, labor market performance and health behavior of the individual during adulthood.

## Methods

### Study Population

Our data sources, SHARE & SHARELIFE were approved by the institutional review board at University of Mannheim, Germany (until 2011) and by the Ethics Council of the Max-Planck-Society for the Advancement of Science (MPG) (2011 onward). The Survey of Health, Ageing and Retirement in Europe (SHARE) has been designed to provide both cross-sectional and life-course data on the health, economic and social conditions of individuals aged 50+ in Europe. The study design is described in detail elsewhere [20,22–24]. The total sample of this study includes 21,127 respondents from 13 countries: Sweden, Denmark, Germany, Netherlands, Belgium, France, Switzerland, Austria, Spain, Greece, Poland and Czech Republic. The respondents in this study participated in the main survey in 2006/7 and then completed a life history interview in 2008/9 (SHARELIFE). Individual life histories provide information on early life circumstances, including parental mental health during childhood, and were collected through a life-grid method with the use of Event History Calendars (EHC) [25,26]. In the EHC the respondent’s life is represented graphically by a grid that is filled automatically in the course of the interview, starting first with life events that are very likely to be remembered accurately. Focusing particularly on SHARELIFE [27] show that the use of the EHC methodology provides a reliable account of early-life conditions. Nevertheless, individuals may still have forgotten certain events or circumstances, especially distant ones. To control for this problem we include a variable which measures delayed memory recall.

A total of 23,678 individuals participated to both the 2006/7 main survey and the life history interview. To focus on a homogenous group, we exclude the cohorts born before 1920 and after 1957 that represent a small share of the sample (948 individuals). The lower bound reflects the fact that the SHARE survey is designed to be representative of the population 50+ (respondents younger than 50 are partners of older individuals). The upper bound excludes the so-called oldest old of whom there are relatively few in the sample because SHARE only focuses on non-institutionalized individuals. We also drop respondents who cannot correctly report the current month and year (409 individuals) as they are likely to suffer from additional psychiatric problems which could have affected their ability to provide data in the survey. In addition, we drop observations for which we have missing values for one of the variables used in the estimations (1,194 individuals, which is only less than 6% of the total sample). This resulted in a study population of 21,127 respondents. The characteristics of the study population are summarized in Table 1.

**Table 1:** Characteristics of the study population*.

VARIABLES	Total (N = 21,127)	Late-life depressive symptoms (N = 5,105)	No late-life depressive symptoms (N = 16,122)
Late-life depressive symptoms	24.16%	100.00%	0.00%
Parental mental health problems during childhood	2.29%	2.72%	2.15%
Age			
- 50–60 years	35.16%	33.89%	35.56%
- 60–70 years	35.03%	31.62%	36.12%
- 70–80 years	22.71%	24.62%	22.09%
- 80+ years	7.10%	9.87%	6.22%
Gender (% Female)	55.24%	70.42%	50.40%
*Childhood circumstances (measured at age 10)*			
Mean (SD) number of rooms per person in the house	0.72 (0.42)	0.65 (0.44)	0.74 (0.41)
Mean (SD) number of facilities in the house (0–5)	1.96 (1.75)	1.60 (1.69)	2.08 (1.76)
Mean (SD) number of books (1–5)	2.09 (1.20)	1.85 (1.13)	2.16 (1.22)
Main breadwinner in low occupation	80.01%	83.84%	78.79%
No father in household	9.08%	10.28%	8.69%
No mother in household	3.70%	4.17%	3.55%
Good school performance in math	33.98%	26.91%	36.24%
Good school performance in language	35.55%	32.26%	36.59%
Health			
- Very good or excellent self-reported childhood health	68.93%	61.94%	71.15%
- One or both parents smoked	62.89%	62.27%	63.08%
- One or both parents drank	8.29%	11.44%	7.28%
*Late-adulthood circumstances*			
Marital status			
- Married	75.02%	68.09%	77.22%
- Divorced	6.72%	7.15%	6.58%
- Widowed	13.42%	19.98%	11.33%
- Never married	4.84%	4.78%	4.87%
Mean (SD) number of children	2.13 (1.39)	2.28 (1.56)	2.09 (1.33)
Socioeconomic status			
- Education			
- Low education	47.73%	58.53%	44.29%
- Medium education	29.63%	26.17%	30.73%
- High education	22.64%	15.30%	24.98%
- Household income (inverse hyperbolic sine transformation)	10.51 (1.39)	10.23 (1.56)	10.60 (1.32)
Health			
- Very good or excellent self-reported health	28.56%	10.64%	34.27%
- at least one ADL limitation	8.06%	18.92%	4.61%
- at least one IADL limitation	13.68%	29.79%	8.55%
Cognitive function:			
- mean (SD) score of numeracy test	3.45 (1.10)	3.01 (1.13)	3.59 (1.06)
- mean (SD) score of delayed recall test	3.66 (1.97)	3.14 (2.00)	3.82 (1.93)
Health Behavior:			
- excessive drinking	7.01%	5.17%	7.60%
- current smoker	19.85%	19.67%	19.91%

*Percentages are presented, unless indicated otherwise. Besides individual and parental smoking, divorced and never married all sub-sample means stratified by current depression state differ significantly from each other based on t-tests with a 5% significance level.

### Late-Life Depressive Symptoms

The SHARE questionnaire includes the EURO-D scale, which was validated in a cross-European study of depression prevalence, EURODEP [28,29]. The scale contains 12 items: sadness or depression, pessimism, wishing death, guilt, sleep, interest, irritability, appetite, fatigue, concentration, enjoyment, and tearfulness. For negatively framed items (e.g., fatigue and pessimism), each symptom is scored 1 if it is present and 0 if it is not. Conversely, for positively framed items (e.g. enjoyment and interest), a symptom is scored 1 if it is absent and 0 otherwise. The items are then summed and clinically significant depression is defined as a EURO-D score greater than 3. This cut-point has been validated in the EURODEP study against a variety of clinically relevant indicators [28,29]. Respondents scoring above this level would likely be diagnosed as suffering from a depressive disorder, for which therapeutic intervention would be indicated [3,28,29].

### Parental Mental Health Problems During Childhood

Information on parental depression during childhood came from the life-history interview of 2008/9. Respondents were asked whether during childhood, defined as the period of life from birth to age 15, any of their parents or guardians had mental health problems or not. While we know lifetime prevalence of mental health problems to be almost 50% [30], in Table 1 we report that 2.29% of our sample report that their parents had some form of mental health problems when the respondent was a child. A potential reason for this discrepancy can be that individuals are more likely to have been aware of and remember severe parental mental health problem; which are much less prevalent than the mild to moderate mental health problems [31] Indeed, while mental health problems in general have an annual prevalence of about 40% in Europe, severe mental health problems are present in about 5% of the working-age population [31]. In that sense we caution the reader to interpret our results as mainly pertaining to impact of severe parental mental health problems during the respondent’s childhood on depressive symptoms in late adulthood.

### Childhood Circumstances

Conditions during childhood refer to when the participant was aged 10 and include 1) the number of rooms per person in the house, 2) the number of facilities (fixed bath, cold running water supply, hot running water supply, inside toilet, central heating; yes/no), 3) the approximate number of books at home (1. none or very few, 2. enough to fill one shelf, 3. enough to fill one bookcase, 4. enough to fill two bookcases, 5. enough to fill two or more bookcases), 4) whether the main breadwinner was in low-skilled occupation, 5) whether the mother and/or the father were not present in the household and 6) whether the respondent had above average school performance in math and in language. We also include measures for childhood health, using a subjective self-reported indicator for whether the respondent enjoyed very good or excellent health between birth and the age of 15, and for parental health behavior, including indicators for whether the parents smoked or drank when the respondent was a child. The internal and external validity of all these childhood indicators has been shown elsewhere [27]. The association of these factors to depression in mid-adulthood has been documented by [14].

### Late-Adulthood Circumstances

Marital status is defined in four categories: married, divorced, widowed and the reference category never married. The number of children is simply a count variable. As indicators of socio-economic status, we use education and household income, measured in natural logarithm to reduce the impact of outliers and adjusted for purchasing power parity to make it comparable across countries. Educational attainment is defined on the basis of the International Standard Classification of Education (ISCED 97): the reference category low education corresponds to lower secondary school or lower (ISCED 0–2), medium education to upper secondary school (ISCED 3) and high education to postsecondary education (ISCED 4–6). Health is assessed using indicators for whether the respondent reports to be in very good or excellent health and for the presence of at least one limitation with activities of daily living (ADL) [32] and with instrumental activities of daily living (IADL) [33]. ADLs include activities such as dressing, walking across a room, bathing or showering and eating. IADLs include using a map to figure out how to get around in a strange place, making telephone calls, taking medications, and managing money. Cognitive function is measured through the score on a numeracy test, assessed by five mathematical calculation tasks, and the score on a delayed recall test, which is given by the number of words recalled (from an initial list of ten words) after an interference period [34]. Measures for behavioral health risk factors include indicators for being currently a smoker and for excessive drinking, defined as drinking more than two glasses of alcohol daily or 5/6 days a week [35]. Individually, each of these factors has been associated to the presence of depressive symptoms among older adults [16,18,19].

### Statistical Analysis

Logistic regression analyses were used to assess the extent to which growing up in a household with a parent with mental health problems is a risk factor toward depressive symptoms in late-life. First, we only include gender, age and country fixed effects, which control for all the unmeasured differences across countries such as institutions and welfare systems. Second, we assess to what extent this association is explained by including controls for childhood circumstances. With the inclusion of additional covariates, the country fixed effects also absorb systematic differences between countries so that the estimated impact of each covariate is the within-country effect of a variation in a specific covariate. Third, we extend the model by adding measures of current late-adulthood circumstances to assess potential pathways by which early-life exposure to a parent with mental health problems translates into depressive symptoms in late-adulthood. Pre-testing revealed that essentially all the various factors included in our specifications are individually significant in univariate regression with and without controls for country, age and gender (results are available on request). We also tested whether the effect of parental mental health on depression differs by gender but we did not find any significant interaction effects (p-values are 0.485, 0.388 and 0.571 for the three specifications in Table 2, respectively). We report odds ratios and 95% confidence intervals. Importantly, while country fixed effects control for unobserved cross-country heterogeneity, it may very well be that the association between current depressive symptoms and parental mental difficulties differs between countries. We return to this issue below when we assess the sensitivity of results to the exclusion of each country separately. Focusing instead on a country by country analysis is infeasible because the number of respondents within each country is insufficient for a thorough statistical analysis. All statistical analyses were performed using Stata 14.

**Table 2:** Multivariate Logistical Regression Analysis.

VARIABLES	OR	95% CI	OR	95% CI	OR	95% CI
Dependent variable:	Depression (EURO-D ≥ 4)					
**Parental mental health problems during childhood**	1.763	(1.429–2.175)	1.540	(1.243–1.908)	1.450	(1.156–1.818)
Age						
- 50–60 years	Reference		Reference		Reference	
- 60–70 years	0.985	(0.908–1.068)	0.940	(0.865–1.022)	0.766	(0.701–0.838)
- 70–80 years	1.260	(1.154–1.376)	1.168	(1.064–1.283)	0.704	(0.632–0.784)
- 80+ years	1.817	(1.602–2.062)	1.747	(1.532–1.993)	0.683	(0.582–0.801)
Female	2.440	(2.276–2.617)	2.388	(2.224–2.564)	2.027	(1.871–2.196)
*Childhood circumstances (measured at age 10)*						
Number of rooms per person			0.857	(0.775–0.947)	0.912	(0.823–1.010)
Number of facilities in the house			0.993	(0.968–1.020)	1.025	(0.997–1.054)
Number of books			0.918	(0.883–0.954)	0.969	(0.930–1.009)
Main breadwinner in low occupation			1.024	(0.928–1.130)	0.944	(0.851–1.048)
No father in household			1.187	(1.051–1.341)	1.130	(0.994–1.285)
No mother in household			1.045	(0.872–1.251)	1.039	(0.860–1.256)
Good school performance in math			0.783	(0.721–0.850)	0.889	(0.815–0.971)
Good school performance in language			1.016	(0.936–1.103)	1.108	(1.016–1.208)
Health						
- Very good or excellent self-reported childhood health			0.675	(0.628–0.726)	0.741	(0.687–0.800)
- One or both parents smoked			1.029	(0.958–1.106)	0.999	(0.926–1.078)
- One or both parents drank			1.554	(1.387–1.741)	1.452	(1.288–1.638)
*Late-adulthood circumstances*						
- Married					0.935	(0.787–1.110)
- Divorced					1.113	(0.902–1.374)
- Widowed					1.194	(0.986–1.446)
- Never married					Reference	
Number of children					1.020	(0.994–1.047)
Socio-economic status						
Education						
- Low education					Reference	
- High education					1.021	(0.908–1.148)
- Medium education					0.958	(0.872–1.052)
- Household income (inverse hyperbolic sine transformation)					0.958	(0.934–0.983)
Health						
- Very good or excellent self-reported health					0.370	(0.334–0.411)
- At least one ADL limitation					2.166	(1.920–2.444)
- At least one IADL limitation					2.423	(2.197–2.672)
Cognitive Function						
- Score of numeracy test					0.836	(0.805–0.868)
- Score of delayed recall test					0.933	(0.914–0.953)
Health behavior						
- Excessive drinking					0.942	(0.808–1.097)
- Smoking					1.119	(1.020–1.227)
country dummies	yes		yes		Yes	
Constant	0.088	(0.074–0.104)	0.175	(0.137–0.224)	0.835	(0.554–1.258)
Observations	21,127		21,127		21,127	
Pseudo R-squared	0.0762		0.0903		0.164	

## Results

The characteristics of the study population are presented in Table 1. Out of all participants, 24% are currently affected by late-life depressive symptoms. Of this group, 2.72% were exposed to parental mental health problems during childhood. In contrast, of the participants who do not currently suffer from late-life depressive symptoms only 2.15% were exposed to parental mental health problems–indicating that an exposure to parental mental health problems may be related to late-life depressive symptoms. In Table 1 we also display sub-sample averages for individuals currently suffering from depression as well as for individuals not currently suffering from depression. This exercise shows that respondents with depressive symptoms late in life are more likely to come from a disadvantaged childhood background and fare less well in late adulthood. Indeed, while of individuals currently displaying depressive symptoms 11.44% grew up with a drinking parent, of individuals who do not currently have depressive symptoms 7.28% grew up with a drinking parent. Comparable results were found for the limitations of Activities of Daily Living (ADL). While 18.92% of individuals currently displaying depressive symptoms had at least one ADL, of those who are not currently displaying depressive symptoms only 4.61% had at least one ADL. As indicated in Table 1, almost all other childhood and late-adulthood circumstances differ significantly depending on the current depression state of the respondent. A correlation matrix of all variables is contained in S1 Table.

The results of the logistic regression analyses are presented in Table 2. The results are divided into three parts. The first, displayed in the first two columns, describes the cross-sectional association between parental mental health problems and depressive symptoms in late adulthood, adjusted for age, gender and country. The second, displayed in the middle two columns, considers how the cross-sectional association is affected when we take into account the childhood circumstances in which the individual was exposed to a parent with mental health problems. The third, displayed in the last two columns, shows how the adulthood environment affects the intergenerational transmissions of mental health problems. We now discuss each of these results in turn.

Individuals who were exposed to a parent with mental health problems during childhood exhibit late-life depressive symptoms more often than those who were not (OR 1.76, 95% CI: 1.43–2.17). With regard to the demographic characteristics, we find that women are much more likely to exhibit depressive symptoms than men (OR 2.44, 95% CI: 2.28–2.61). Compared to the reference group (age 50–60), individuals aged 70–80 exhibit a higher probability of experiencing depressive symptoms (OR 1.26, 95% CI: 1.15–1.38). This effect increases further with age so that those aged 80 and over experience the most depressive symptoms (OR 1.82, 95% CI: 1.60–2.06). We briefly return to this point below.

### Childhood Circumstances

Taking into account the shared environment of the child and the parent is seen to reduce the intergenerational transmission of mental health problems with the OR now being 1.54 (95% CI: 1.24–1.91). Of the shared environment a number of such factors stand out. For instance, individuals of whom either one of the parents drank are much more prone to exhibit depressive symptoms (OR 1.55, 95% CI: 1.39–1.74). Moreover, growing up without a father present is associated with an elevated chance of exhibiting depressive symptoms (OR 1.19, 95% CI: 1.05–1.34).

### Late-Adulthood Circumstances

Focusing on the late-adulthood environment reduces the intergenerational transmission of mental health problems further (OR 1.45 95% CI: 1.16–1.82). Amongst others, individuals with bad self-reported health and/or ADL limitations are at risk for developing depressive symptoms (OR 2.70, 95% CI: 2.43–2.99 & OR 2.17, 95% CI: 1.92–2.44). Interestingly, after taking into account the various late-adulthood health and lifestyle factors, the positive relationship between age and depression documented above becomes negative with an ORs of 0.77 (95% CI: 0.70–0.84), 0.70 (95% CI: 0.63–0.78) and 0.68 (95% CI: 0.58–0.80) for age groups 60–70, 70–80 and 80 and over, respectively.

## Discussion

Our results show that there is a positive association between depressive symptoms in late adulthood and parental mental health problems during childhood. This association is attenuated after adjusting for childhood and late-adulthood circumstances. Our results are in line with previous research [14] who, using the 1970 British Cohort Study, also found a positive relationship (corr. 0.190) between maternal mental health and individual mental health in mid-adulthood (age 34). Moreover, they show that the magnitude of the relationship declines when they take into account the shared environment in which the child grew up (corr. 0.163). Taking into account also the health of individuals in adulthood further reduces their documented relationship between maternal and individual mental health (corr. 0.133).

However, our study focusses on the occurrence of depression among late-adults and investigated the association between parental mental health problems and depression in late-adulthood and to what extent this association is influenced by life-course socio-economic, health and lifestyle factors. To our knowledge, this has never been investigated before. Moreover, while [14] focus on a narrowly defined sample (i.e., a sample of individuals born between April 4–11 1970 in Great Britain), we consider individuals currently 50+ from all over Europe from many different birth years. This allows us to extend the current state of the literature in a number of important directions. First of all, we show that the general pattern of the analysis is the same for late-adults. Second, the geographical scope of our analysis is much wider and covers a larger number of relatively diverse western European countries. Third, we show that the association between depressive symptoms in late-adulthood and parental mental health difficulties extends to cohorts born between 1920 and 1957.

Earlier literature suggests that depression among late adults is currently not sufficiently recognized and, therefore undertreated [36]. In that sense, our paper contributes to the recognition of the ways along which sharing an environment with a parent having mental problems translates into depressive symptoms in late adulthood. Hereby, we aim to pave the way toward care-giving strategies aimed at ameliorating the consequences of exposure to parents with mental health problems.

## Limitations

Due to missing data we had to drop 5.6% of observations from our analysis. We compared the characteristics of those with complete data and those with any missing data. The prevalence of depression is slightly higher in the sample with any missing data (26.13%) than in the sample with complete data (24.16%) but the difference is not statistically significant (p-value = 0.1321). As for our main explanatory variable, 2.10% of those with missing data and 2.29% of those with complete data were exposed to parental mental health problems during childhood. A formal test shows also in this case that the difference is not statistically significant (p-value = 0.668). The results of these tests, together with the low proportion of missing values, lead us to believe that our results are not seriously biased.

The second limitation is that we cannot perform our analysis on a country-by-country basis due to small sample sizes on a country level. Such an analysis could potentially provide insights into how the associations we document above differ between countries. In lieu of such an analysis we assess the sensitivity of our findings to the exclusion of each country one-by-one. This exercise is portrayed in the S2 Table and shows that the association, and its attenuation due to childhood and late-adulthood circumstances, are not much affected by the exclusion of any country. Hence, we are confident that it is not a single country that is driving all our results.

A further limitation of our study is that we rely on self-reported accounts of childhood circumstances of individuals currently aged 50+. While the survey design of SHARE carefully aimed to limit the potential impact of recall bias, it may still be that certain individuals are unable to provide a precise account of their childhood circumstances. To ameliorate this problem we include a covariate measuring delayed memory recall. Naturally, this may not control for the full magnitude of the problem. In terms of our exposure variable this can imply that parental mental health difficulties may be of a particularly severe nature for the respondent to remember it after so many years. As discussed above, this suspicion is supported by the discrepancy between epidemiological data on mental health problems and the prevalence of parental mental health problems in our sample. This means that our results are probably best seen as the association between rather severe parental mental health difficulties and depressive symptoms in late adulthood–milder mental health difficulties may show a different pattern of association.

Third, related to the previous point, we rely on self-reported accounts of parental mental health. Therefore the measure may not capture true mental health problems but only observed ones. Indeed, it may be that a parent was suffering from mental health problems but was successful in managing it in such a way that the child never felt sufficiently exposed so as to report on it when asked in the questionnaire. In that sense, it is likely that the mental health problems of the parents as reported by the respondents are severe mental health problems. Moreover, individuals may report that their parents suffered from mental health issues in order to rationalize their own struggle with mental health problems. Such justification bias can potentially lead the estimated associations to be overestimates of the true associations. It is not, however, evident how such attribution error influences the attenuation of the association due to the inclusion of childhood and late-adulthood characteristics in the estimation specification. An additional limitation arises from the fact that we do not know which parent or guardian was affected by mental health problems.

Fourth, even though SHARE and SHARELIFE provide us with a broad array of childhood and late-adulthood characteristics, a number of known outcomes of parental mental illness and potential pathways of the intergenerational transmission of mental health problems where not measured. Therefore our analysis was not able to study how variables such as child abuse and neglect, insecure attachment and poor parenting influence the association between childhood parental mental health difficulties and depressive symptoms in late adulthood.

Finally, the fact that, to the best of our knowledge, ours is the first analysis to consider the association between childhood parental mental health difficulties and depressive symptoms in late adulthood using a large cross-national retrospective survey raises novel research questions that in sake of space and scope could not be addressed. Future research could, for instance, consider how the accumulation of various risk or protective factors during childhood affects depression in late adulthood [37]. In a similar vein, future research could proceed with a more formal mediation analysis to identify which of our childhood and/or late-adulthood characteristics could be considered as moderators, which as mediators and which as common risk factors.

## Conclusions & Recommendations

To our knowledge, this study is the first study that investigated how the association between depressive symptoms in late-adulthood and parental mental health difficulties is influenced by life-course socio-economic, health and life-style factors. Individuals whose parents displayed mental health problems are more likely to exhibit depressive symptoms in late-adulthood. The childhood as well as adulthood environments attenuates some of this relationship.

Our results emphasize the need to invest in effective intervention and prevention strategies to ameliorate the burden of disease from depression from both the individual as well as the collective perspective. In this regard an important starting point is to improve the recognition and acceptance of mental health problems among the general population and late adults in particular [36]. More specifically, two recommendations stand out. First of all, effective treatment of mental health problems should focus on the patient as well as his/her offspring as doing so may potentially break the intergenerational transmission of mental health problems [38,39]. Second, in designing intervention and prevention strategies, our results suggest that besides direct treatment of health problems, policy makers and practitioners should focus on potentially amendable environmental factors. In childhood such factors include the school performance and the drinking behavior of the parents. In adulthood measures to boost household income and reduce adverse health behavior such as smoking are potential policy targets.

## Supporting Information

S1 Table Correlation matrix. (XLSX)

S2 Table Excluding one country at a time. (XLSX)
